# Double and Single True Knot of an Umbilical Cord: A Case Report

**DOI:** 10.7759/cureus.36393

**Published:** 2023-03-20

**Authors:** Jane E Waldron, Sean M Muir, James Hubbard

**Affiliations:** 1 Department of Medicine, Edward Via College of Osteopathic Medicine, Spartanburg, USA; 2 Department of Obstetrics and Gynecology, Piedmont Medical Center, Rock Hill, USA

**Keywords:** umbilical cord abg, nuchal cord, placenta, umbilical cord, double true knot, true knot

## Abstract

The primary function of the umbilical cord is to transport blood to and from the fetus. It carries deoxygenated blood away from the fetus by two umbilical arteries, and oxygenated blood from the placenta toward the fetus by an umbilical vein. In some cases, the umbilical cord can form a true knot increasing the risk of asphyxia and fetal demise. The umbilical cord may also form a false knot, which is only a kink and will not increase fetal risk of abnormalities.

A 40-year-old woman, gravida six, parity three (G6P3), presented to the hospital in active labor after 39.1 weeks of gestation. Six hours after admission a healthy male fetus was delivered with one nuchal cord. The placenta was delivered approximately 3 minutes later. Upon inspection, the presence of a double and a single true knot of the umbilical cord was noted. This case describes a fetus with a double and single true knot of the umbilical cord that was not apparent by ultrasonography.

## Introduction

The umbilical cord develops at week 3 of gestation from a connecting stalk and fuses with the omphalomesenteric duct. By week 7, full development generally occurs [1]. The umbilical cord is composed of two umbilical arteries, one umbilical vein, vitelline duct, and Wharton’s jelly, all surrounded by an amniotic membrane. The main function of the umbilical cord is to serve as a conduit to transport blood to and from the fetus. The two umbilical arteries carry deoxygenated blood away from the fetus toward the placenta, while the umbilical vein carries oxygenated blood from the placenta back to the fetus [1]. Elongation​ of the cord occurs during the second trimester. The average umbilical cord dimensions are 40-50 cm in length and 2 cm in diameter, with approximately 40 helical turns [1,2].

Umbilical cord abnormalities include morphologic and placental insertion alterations, in utero distortion, number of vessels, blood flow patterns, and cystic and solid/complex masses. In utero distortion abnormalities include cord knot, cord torsion, nuchal cord, and cord entanglement (if monoamniotic twins) [3-5]. Umbilical cord knots usually present when the umbilical cord forms a loop that tightens down on itself and interlaces. Most umbilical cord knots occur between nine and 12 weeks, however, some research has suggested that knots can occur during labor. Three types of knots are reported in the literature, a true knot, a false knot, and a loose knot. A false knot is a kink in the umbilical cord that is not clinically significant. True knots are tightened before the onset of labor as opposed to a loose knot. The incidence of a true umbilical cord knot ranges from 0.3-2% with increased incidences reported in polyhydramnios, small fetus size, gestational diabetes mellitus, and male fetuses [2,4]. Complications of true umbilical knots include decreased umbilical cord blood flow, fetal asphyxia, and death [4]. Most knots are diagnosed by ultrasound; however, knots can be easy to miss, which leads to increased risk of fetal complications. Management is dependent upon knot tightness and monitoring fetal heart tones [6]. Any indication of fetal hypoxia indicates the need for a cesarean section (C-section).

## Case presentation

**Figure 1:**
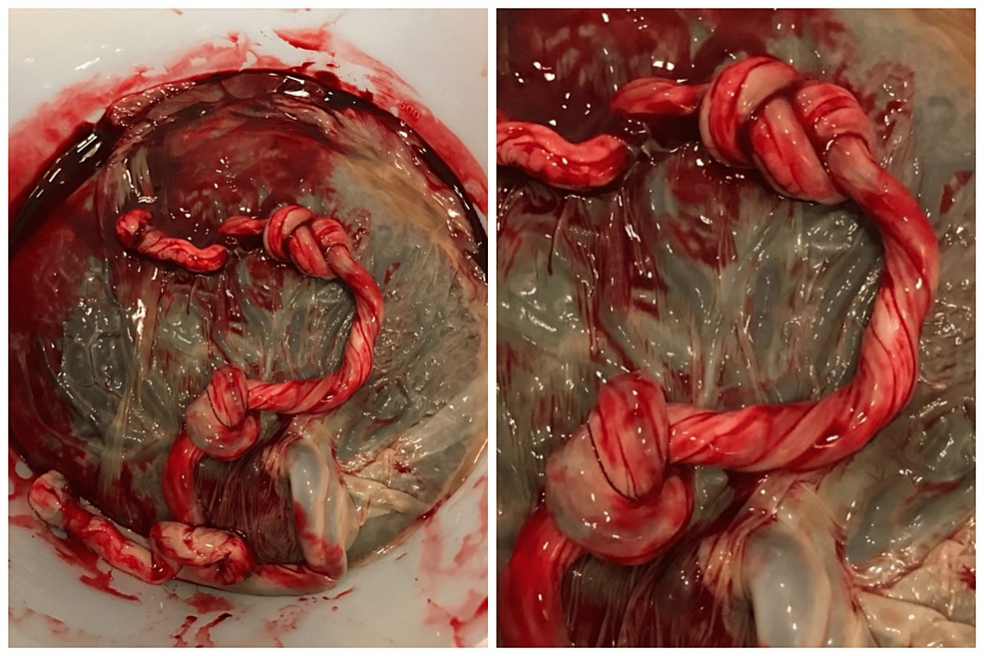
Double and single true knot of an umbilical cord. A 39 x 1 cm placenta delivered approximately three minutes after the delivery of a newborn male. The first knot was located 30 cm from the insertion, the second knot, a double knot, was 38 cm from the insertion site. Meconium staining was noted around the edge of the placenta.

A 40-year-old woman, G6P3, in active labor was admitted to the hospital after 39 weeks gestation. Routine ultrasound examination was performed at 12 and 19 weeks. Both evaluations were normal. The antenatal course was notable for advanced maternal age, Herpes Simplex Virus, and an abnormal 1-hour glucose tolerance test. A 3-hour glucose tolerance test was within normal limits. Upon admission, cardiotocography was initiated and showed a fetal heart rate of approximately 140 beats per minute with a category 1 tracing for the entirety of labor. Six hours after admission, a viable 3190 g male neonate was delivered with one nuchal cord and moderate meconium. Apgar scores at 1 and 5 minutes were 8 and 9, respectively. The placenta was delivered approximately 3 minutes later. Upon inspection of the umbilical cord, the presence of a double and a single true knot was noted. The arterial blood gas of umbilical vein blood was pH of 7.314, pCO2 47.4 mmHg, and pO2 <39.4 mmHg. Pathological examination revealed a 432 g placenta (weight between 10th and 25th percentile for gestational age) with a 39 x 1 cm three vessel umbilical cord and a true knot located 30 cm from the insertion site and a second knot 38 cm from the insertion site (Figure 1). The baby appeared normal and healthy, was admitted to the neonatal nursery and was discharged two days later.

## Discussion

The umbilical cord performs a vital function of transporting nutrients to and from the fetus and discarding waste products. The umbilical cord serves to transport oxygenated blood from the placenta to the fetus and deoxygenated blood from the baby to the placenta through the umbilical vein and umbilical artery, respectively. The most common umbilical cord anomaly is umbilical cord prolapse. Cord prolapse commonly results in stillbirth [2,4]. Other complications of the umbilical cord include abnormal morphology, placental insertion, in utero distortion, number of vessels (too few or too many), blood flow pattern, and cystic and solid/complex masses [2,4,5]. The epidemiology of a single true knot is 0.5-2% and therefore both a double and a single true knot are less than 0.5%. Most single true knots increase the risk of fetal mortality, intrauterine fetal demise, and stillbirth.

This case presents a double and single true knot of the umbilical cord that did not result in fetal physical or neurological abnormalities or death.

The prognostic implications of a true knot are dangerous and therefore early diagnosis is essential. Doppler-assisted ultrasonography is the current gold standard for diagnosing umbilical abnormalities. Sepulveda et al. investigated 5575 deliveries in which 18 newborns had a true knot. None of the 18 fetuses were noted to have a true knot during the second-trimester ultrasound examination [6]. Color Doppler ultrasonography at the third trimester did not identify a knot in 13 out of the 18 fetuses. Historically, the “hanging noose sign” or “four-leaf-clover sign” are documented confirmatory images for the diagnosis of a true knot during color Doppler ultrasonography or power (i.e., low frequencies, high sound intensity) ultrasonography. However, most knots are misdiagnosed indicating that improved diagnostic technologies or techniques need to be developed [5-7]. The percentage of knots missed by ultrasonography should also be investigated.

Some physicians believe that knots do not form during development, but intrapartum. Therefore, it may be beneficial to use Doppler ultrasonography screening techniques and positional maneuvers prior to delivery to diagnose a true or loose knot. Additionally, loose knots do not always present as “hanging noose” or “four-leaf-clover” signs, and may be missed thus leading to fetal or delivery complications if a loose knot progresses into a true knot [5-7]. One of the barriers to diagnosing a knot is that it is unknown when and how they occur. The most popular and reasonable hypothesis is that fetal movement leads to twisting of the umbilical cord resulting in a half knot or true knot. This correlates with risk factors; polyhydramnios, male fetuses, and gestational diabetes. Other risk factors include long umbilical cord, advanced maternal age, and multiparity. Our patient was noted to have advanced maternal age, multiparity, a male fetus, and gestational diabetes possibly leading to a smaller baby and increased risk of knots.

Expectant management and monitoring of fetal heart tones is the current standard of care if a true knot is diagnosed by ultrasound prior to labor. If signs of fetal distress are diagnosed, a C-section is warranted. Induced vaginal deliveries increase the risk of loose knots becoming true knots, or asymptomatic true knots becoming symptomatic true knots [4]. This suggests that physicians should perform a C-section in deference to vaginal delivery. The sequence of events based upon fetal interrogation (i.e., vaginal delivery or C-section) should be discussed and agreed upon prior to hospital admission for labor to reduce complications.

## Conclusions

True knots of the umbilical cord are difficult to diagnose and can result in fetal asphyxia, decreased fetal blood flow, and increased fetal death. This case presents a double and single true knot of the umbilical cord that was not apparent on ultrasound. As technology continues to advance, the early diagnosis of umbilical cord knots will allow us to follow their clinical course so we can come to better understand how to manage these pregnancies. By monitoring through ultrasound techniques, complications could potentially be prevented or recognized earlier. If a true knot is diagnosed, continued imaging and close observation until fetal maturity is reached gives the best chance for reduced complications.
